# An NMR-based approach reveals the core structure of the functional domain of SINEUP lncRNAs

**DOI:** 10.1093/nar/gkaa598

**Published:** 2020-07-22

**Authors:** Takako Ohyama, Hazuki Takahashi, Harshita Sharma, Toshio Yamazaki, Stefano Gustincich, Yoshitaka Ishii, Piero Carninci

**Affiliations:** NMR Division, RIKEN SPring-8 Center (RSC), RIKEN, 1-7-22 Suehiro-cho, Tsurumi-ku, Yokohama, Kanagawa 230-0045, Japan; Laboratory for Transcriptome Technology, RIKEN Center for Integrative Medical Sciences, Yokohama, Kanagawa 230-0045, Japan; Laboratory for Transcriptome Technology, RIKEN Center for Integrative Medical Sciences, Yokohama, Kanagawa 230-0045, Japan; NMR Division, RIKEN SPring-8 Center (RSC), RIKEN, 1-7-22 Suehiro-cho, Tsurumi-ku, Yokohama, Kanagawa 230-0045, Japan; Central RNA Laboratory, Instituto Italiano di Tecnologia (IIT), 16163 Genova, Italy; NMR Division, RIKEN SPring-8 Center (RSC), RIKEN, 1-7-22 Suehiro-cho, Tsurumi-ku, Yokohama, Kanagawa 230-0045, Japan; School of Life Science and Technology, Tokyo Institute of Technology, 4259 Midori-ku, Yokohama, Kanagawa 226-8503, Japan; Laboratory for Transcriptome Technology, RIKEN Center for Integrative Medical Sciences, Yokohama, Kanagawa 230-0045, Japan

## Abstract

Long non-coding RNAs (lncRNAs) are attracting widespread attention for their emerging regulatory, transcriptional, epigenetic, structural and various other functions. Comprehensive transcriptome analysis has revealed that retrotransposon elements (REs) are transcribed and enriched in lncRNA sequences. However, the functions of lncRNAs and the molecular roles of the embedded REs are largely unknown. The secondary and tertiary structures of lncRNAs and their embedded REs are likely to have essential functional roles, but experimental determination and reliable computational prediction of large RNA structures have been extremely challenging. We report here the nuclear magnetic resonance (NMR)-based secondary structure determination of the 167-nt inverted short interspersed nuclear element (SINE) B2, which is embedded in antisense Uchl1 lncRNA and upregulates the translation of sense Uchl1 mRNAs. By using NMR ‘fingerprints’ as a sensitive probe in the domain survey, we successfully divided the full-length inverted SINE B2 into minimal units made of two discrete structured domains and one dynamic domain without altering their original structures after careful boundary adjustments. This approach allowed us to identify a structured domain in nucleotides 31–119 of the inverted SINE B2. This approach will be applicable to determining the structures of other regulatory lncRNAs.

## INTRODUCTION

Comprehensive mouse transcriptome analysis has identified a large number of long non-coding RNAs (lncRNAs) containing retrotransposon elements (1). Short interspersed nuclear elements B2 (SINE B2) are rodent-specific retrotransposon elements derived from transfer RNAs, with 450 000 copies currently annotated in the mouse genome (2–6). Despite the large copy numbers of SINE B2 elements, their biological roles are still poorly understood because of the sequence divergence that has occurred during evolution (7). Several studies have demonstrated that non-coding RNAs transcribed from SINE B2 regions specifically inhibit RNA polymerase II transcriptional activity by binding of a key domain of the SINE B2 RNA secondary structure to the polymerase (8,9).

Our previous study has revealed that antisense ubiquitin carboxy-terminal hydrolase L1 (AS-Uchl1) lncRNA contains a 167-nt inverted SINE B2, which is an effector domain that stimulates the translation of sense Uchl1 mRNA (10). This finding has yielded various applications for upregulating other mRNA targets; synthetic AS lncRNAs, named SINEUPs (as SINE elements that upregulate translation) are comprised of a binding domain—a complementary sequence of target mRNAs—and an effector domain consisting of the SINE B2. SINEUPs broadly enhance the translation of targeted mRNAs (11–17). Protein translation stimulation by SINEUPs is in stark contrast to previous findings suggesting a transcription inhibitory role for SINE B2 RNAs (18). In addition to mouse SINE B2, we also found that human *Arthrobacter luteus* (Alu) elements (FRAM and MIRb) enhance target mRNA translation, although the sequence identity among these RNAs is <50% (14). We hypothesize that the identity of SINE RNA structures, rather than their sequence identity, plays a key role in translational stimulation. To test our hypothesis, an initial detailed understanding of a fragment of a SINEUP RNA structure was achieved, confirming the associations between SINEUP structure and function (11,19). To estimate the secondary structure of inverted SINE B2, we previously used chemical probing by dimethyl sulfate and 1-cyclohexyl-(2-morpholinoethyl) carbodiimide metho-*p*-toluene sulfonate, with secondary structure prediction using information on unpaired bases detected by the chemical probes as restraints (19). This approach does not offer experimental evidence of the pairing of specific bases, or of the stability of base-pairing, or any information on the tertiary structure. We therefore used nuclear magnetic resonance (NMR) spectroscopy to further determine the secondary and tertiary structures of the 38-nt RNA fragment that NMR analysis has revealed is essential for inverted SINE B2 regulatory function (19). However, *de* *novo* structural analysis of a full-length lncRNA or its embedded long functional RNA element has been extremely challenging. As of June 2019, about 1400 RNA structures (including those of short RNAs) were registered in the Protein Data Bank (PDB), but only 157 structures of long RNAs (>100 nt). More than 140 000 structures are registered for proteins in the PDB (20) (http://www.rcsb.org/). Also, with the exception of one case (21), no structures of lncRNAs or long RNA elements of multi-domain lncRNAs exceeding 200 nt have been deposited in the PDB. Only a handful of crystallography and NMR studies have revealed the structures of non-coding (nc)RNAs with 50 to 120 nt; many of these ncRNAs are in complexes with RNA-binding proteins (RBPs) (22–24). Generally, long RNAs are difficult to crystalize and are often too dynamic for cryo-electron microscopy studies. Although NMR has great potential for use in studying the structure of lncRNAs in solution, structural determination of RNAs is generally limited to those with molecular weights of up to ∼15 k (∼45 nt); the longest RNA structure determined by NMR was that of a 155-nt RNA (25) and a few other NMR-based ncRNA structures exceed the limit of ∼100 nt (26–29). Furthermore, NMR-based methods of structural determination of nucleic acids remain underdeveloped compared with those for proteins. Therefore, reliable experimentally based secondary structures are scarce for lncRNAs, despite the fact that information on secondary structures is essential to the structural determination of RNAs by NMR.

Here, we demonstrated a systematic approach to determine the structure and the functional domains of a 167-nt (MW ∼55 k) functional inverted SINE B2 RNA, for which there is little structural information, by using NMR. First, we prepared fragments to identify the region responsible for the structure relevant to the SINEUP function. Second, we compared the ^1^H NMR spectra of fragmented inverted SINE B2 with the deletion of *n* × 10 nt from both the 5′ end and the 3′ end. The deletion experiments suggested that inverted SINE B2 includes at least two structured domains, which we later named the C and M domains. Third, we compared our NMR findings with five secondary structures calculated by prediction programs. Two of them were consistent with the NMR experimental data and were used as reference structures. To determine the secondary structure of inverted SINE B2, we divided it into four fragments. Finally, the whole secondary structure was determined, with the assignment of most of the imino proton signals of the 167 nt of full-length inverted SINE B2. Interestingly, our NMR experiments revealed that the terminal domain (T domain) in nucleotides 1–21 and 141–167—for which all of the programs predicted a stem structure—did not form a rigid stem. The 31–119 fragment (largely corresponding to the central domain (C domain) in nucleotides 34–119), which retained 80% of the SINEUP function of the full-length inverted SINE B2, showed an identical fold to that of the corresponding region of the full-length RNA. This suggested that U118 and G119 are important for the stability of the region 43–58 stem-loop structure. We also identified a stable stem formation in the middle domain (M domain) in nucleotides 23–33 and 130–140. Thus, we identified two structured domains (C and M domains) and one dynamic domain (T domain), the structures of which deviated substantially from any of previous secondary structure predictions. Our NMR results, together with those of previous deletion experiments, suggest that the 43–58 stem-loop may include a tertiary structure and is vital to the regulatory role of SINEUPs (11,19). Our strategy for determining secondary structure can be widely applied to other ∼ 200-nt functional RNAs.

## MATERIALS AND METHODS

### Plasmid and cloning

Δ5–14, Δ35–44, Δ65–74, Δ100–111, Δ100–111 Δ130–141, Δ130–141, and Δ1–30 Δ120–167 deletion mutants (fragment 31–119) were created by using molecular cloning techniques. Briefly, fragment 31–119 (Δ1–30 Δ120–167) was chemically synthesized (Cell Guidance Systems, Cambridge, UK) and swapped with the full-length inverted SINE B2 sequence in SINEUP-GFP (Δ5′–32 nt); Δ5–14, Δ35–44, Δ65–74, Δ100–111, Δ100–111 Δ130–141 and Δ130–141 deletion mutants were mutagenized by using QuikChange II site-directed mutagenesis kits (Agilent Technologies Inc., Santa Clara, CA, USA) in accordance with a protocol published previously (11,30).

### Cell culture and transfection

Full-length inverted SINE B2 plasmid (SINEUP-GFP (Δ5′–32)) and the fragment 31–119 plasmid (see ‘Plasmid and cloning’ section) were transfected with pEGFP-C2 plasmid (Takara Bio US, Inc., Mountain View, CA, USA) into HEK293T/17 (human embryonic kidney) cells (ATCC: CRL-11268) in accordance with a protocol published previously (11,30).

### Protein extraction and Western blot

Cells were lysed with 10% cell lysis buffer (Cell Signaling Technology, Denver, CO, USA) and 0.005% phenylmethylsulfonyl fluoride (PMSF) (Cell Signaling Technology), and then the protein lysates (10 μg) were separated by 10% polyacrylamide gel electrophoresis (TGX precast protein gels, Bio-Rad, Hercules, CA, USA). Green fluorescent protein (GFP) and β-actin proteins were detected by western blotting, which was performed in accordance with a protocol published previously (11,30). GFP fold change was calculated from the band intensity of GFP after normalization against the band intensity of an internal control (β-actin). All calculations were performed by using ImageJ software (https://imagej.nih.gov/ij/), as described in references (11) and (30).

### Antibodies

Enhanced green fluorescent protein (EGFP) was detected by using anti-GFP rabbit antibody (Thermo Fisher Scientific, Waltham, MA, USA, Cat. no. A-6455). β-Actin was detected by using anti-β-actin antibody (Sigma-Aldrich, St. Louis, MO, USA, Cat. no. A5441,). The secondary antibodies were horseradish peroxidase (HRP)-conjugated polyclonal goat anti-rabbit antibody (DAKO, Glostrup, Denmark, Cat. no. P0448) and HRP-conjugated polyclonal goat anti-mouse antibody (DAKO, Cat. no. P0447).

### Total RNA extraction, reverse transcription (RT) reaction and quantitative RT-PCR

Total RNA was extracted by using a Maxwell RSC simplyRNA Cells Kit (Promega, Fitchburg, WI, USA) and Maxwell RSC Instrument in accordance with the manufacturer's protocol. Total RNA (500 ng) was reverse transcribed to cDNA by using a PrimeScript 1st strand cDNA synthesis kit (TaKaRa Bio Inc., Shiga, Japan) in accordance with the manufacturer's protocol. SINEUP-GFP RNA, EGFP RNA and GAPDH RNA expression were quantified by polymerase chain reaction (PCR) amplification using SYBR Premix Ex Taq (Tli RNaseH Plus) (TaKaRa Bio Inc.). Relative expression was analyzed by using the 2^−ΔΔCT^ method (31).

The following qRT-PCR primers were used:

hGAPDH_Fw: TCTCTGCTCCTCCTGTTC
hGAPDH_Rv: GCCCAATACGACCAAATCC
EGFP_Fw: GCCCGACAACCACTACCTGAG
EGFP_Rv: CGGCGGTCACGAACTCCAG
SINEUP-GFP_Fw: CTGGTGTGTATTATCTCTTATG
SINEUP-GFP_Rv: CTCCCGAGTCTCTGTAGC.

### Secondary structure prediction programs

All predicted secondary structures were calculated by using the user-defined parameters of the computational prediction software on the respective web servers. The temperature was set to 37°C (310.15K) for all predictions. All predictions were performed under standard parameters, including the energy model recommended by each program (in cases where selection from among several models was permitted). For Mfold (32), ‘RNA sequence’ was linear, with 1 M NaCl and no divalent ions; the percentage suboptimality value was set to 5, the ‘maximum interior/bulge loop size’ was set to 30 and a ‘maximum interior/bulge loop size’ of 30 was selected (32). For RNAfold, the options ‘minimum free energy’ and ‘partition function’ and ‘avoid isolated base pairs’ were selected (33). A centroid secondary structure was chosen. For RNAstructure, we selected a maximum loop size of 30, maximum % energy difference of 10, maximum number of structures 20, window size 3, gamma 1, iteration of 1 and minimum helix length of 3 were selected (34). For Vfold2D, Turner parameters (04 version) were selected (35). For Vsfold5, the polymer model options were ‘Jacobson-Stockmayer’, ‘Kuhn-length 9’ (a typical value for functional RNAs), ‘search for pseudo-knots’, a minimum linkage stem length of 5, a leading edge length of 7 and no inclusion of Mg++ (36).

### DNA templates for *invitro* RNA transcription

Template DNAs for the inverted SINE B2 fragments (nucleotides 31–117, and domains TM and MC2) were purchased from Eurofins Genomics (Tokyo, Japan) and Hokkaido System Science Co., Ltd. (Hokkaido, Japan) The DNA template of full-length inverted SINE B2 (CAGTGCTAGAGGAGGTCAGAAGAGGGCATTGGATCCCCCAGAACTGGAGTTATACGGTAACCTCGTGGTGGTTGTGAACCACCATGTGGATGGATATTGAGTTCCAAACACTGGTCCTGTGCAAGAGCATCCAGTGCTCTTAAGTGCTGAGCCATCTCTTTAGCTCC) for *in vitro* transcription was amplified from SINEUP-GFP (10) by using vector backbone pcDNA3.1 plasmid (Thermo Fisher Scientific) and PCR with Tks Gflex DNA polymerase (Takara Bio Inc.).

Five forward primers (1: TAATACGACTCACTATAGGCAGTGCTAGAGGAGGTCAGAAGAG; 11: TAATACGACTCACTATAGGAGGTCAGAAGAGGGCAGG; 21: TAATACGACTATAGGAGAGGGCATTGGATCCCC; 31: TAATACGACTCACTATAGGATCCCCCAGAACTGGAGTTATA; 41: TAATACGACTCACTATAGGAACTGGAGTTATACGGTAACCTC) and eight reverse primers (117: GGACCAGTGTTTGGAACTCAATATC; 118: AGGACCAGTGTTTGGAACTCAATATCC; 119: CAGGACCAGTGTTTGGAACTCAATATC; 127: CTCTTGCACAGGACCAGTGTTTG; 137: GCACTGGATGCTCTTGCACAG; 147: GCACTTAAGAGCACTGGATGCTCTT; 157: AGATGGCTCAGCACTTAAGAGCACT; 167: GGAGCTAAAGAGATGGCTCAGCACT) were purchased from Eurofins Genomics. A T7 promoter sequence (forward: TAATACGACTCACTATA) was added to each primer sequence. The amplified templates were purified twice by ethanol precipitation and then lyophilized.

### *In vitro* RNA transcription and RNA purification

All RNA fragments were transcribed *in vitro* by using T7 RNA polymerase from an AmpliScribe T7-Flash Transcription Kit (Lucigen, Madison, WI, USA) and precipitated by using isopropanol with 2 mM ethylenediaminetetraacetic acid (EDTA). RNA pellets were suspended in 8 M urea, 0.1% Orange G, 0.05% xylene cyanol and 0.05% bromophenol blue running dye, and then centrifuged at 20 000 × *g* for 15 min at room temperature. The supernatants, which contained the RNAs, were separated in a 12.5% polyacrylamide—8 M urea gel. After the electroseparation, the appropriate bands were cut out and the RNAs were collected by using an electro-elution system (Model 422 Electro-Eluter, Bio-Rad). The RNAs were collected by ethanol precipitation with 2 mM EDTA twice. RNA pellets were dissolved in MilliQ water (MerckMillipore, Darmstadt, Germany) and then lyophilized.

### NMR spectroscopy

Four buffers were tested the NMR: 20 mM sodium phosphate, 50 mM NaCl (pH 7.2 or pH 6.85); 20 mM 2-(N-morpholino)ethanesulfonic acid (MES), 50 mM NaCl (pH 6.0); and 89 mM Tris-borate (pH 8.9). The pH of the MES buffer was adjusted to 6.0 with NaOH. All buffers contained 8% D_2_O.

For structure determination, RNAs were dissolved in 20 mM MES—NaOH (pH 6.0), 50 mM NaCl and 8% D_2_O, with the solution then adjusted to 30–300 μM. Shigemi tubes (diameter 4 mm) (Shigemi, Tokyo, Japan) were used for the full-length inverted SINE B2 RNA and 31–119 RNA samples, and 5-mm-diameter Shigemi tubes were used for the other RNAs. It was found that samples prepared and kept in the NMR tubes showed no NMR spectral changes for at least a year without the addition of RNase inhibitors. We confirmed that the NMR spectra of the full-length inverted SINE B2 or its fragments did not change with the addition of an annealing protocol. Therefore, all of the NMR spectra used in this work were recorded from samples without annealing.

NMR spectra were recorded on Bruker Avance 700, 800 and 900 MHz spectrometers with cryogenic probes (Bruker Biospin, Inc., Billerica, MA, USA). 1D ^1^H NMR spectra were collected with a data size of 16 k, 512 scans and a spectral width of 25 ppm at 298K. 2D NOESY (nuclear Overhauser effect spectroscopy) experiments were performed with 2048 × 512 data points and a spectral width of 25 ppm at 288K or 293K. Water suppression was achieved with a Watergate 3–9–19 pulse sequence. Spectra were analyzed by using Sparky NMR assignment and integration software (https://www.cgl.ucsf.edu/home/sparky/) and TopSpin 3.61 NMR processing software (Bruker Biospin, Inc.).

### Optimization of NMR buffer

Sodium phosphate is a standard NMR buffer used for biological samples, especially in the case of proteins. The principal advantages of using phosphate buffer for NMR measurements are threefold: a phosphate buffer provides no detectable proton, it is stable at neutral pH and little pH change is introduced by temperature changes. However, in the case of RNA, sodium phosphate is often not adequate because the pH range of phosphate buffer is a little higher than the optimal pH for detecting imino proton signals. The ^1^H exchange rate between the imino group and water depends on the solvent pH: exchange is minimized at pH lower than 5. Moreover, the thermodynamic stability of some functional RNAs is influenced by the solvent pH: for example, in the case of tRNA^Lys,3^, the *T*_m_ value increases by about 6°C at pH 5 compared with pH 7.2 (37). However, acidic conditions with pH lower than 5 may induce structural changes by protonation of the amides of bases (38). The optimal pH range of sodium phosphate buffer is neutral to weakly alkaline (pH 7–9), but, as noted above, the exchange rate between the imino group and water is minimized at pH lower than 5. To focus on the observation of imino proton signals by NMR while minimizing artifacts, we considered that weak acid buffer at pH >5 would be more suitable than neutral buffers such as sodium phosphate. We therefore prepared four buffers, namely sodium phosphate (pH 7.2 and pH 6.85), MES-NaOH (pH 6.0) and Tris-borate (pH 8.9). MES was selected for two reasons: the ^1^H NMR signals of this compound do not overlap with those of most RNA signals, and the optimal pH of MES buffer solutions includes a weak acid range. Tris-borate was selected as another weak alkaline buffer for negative control. It should be also noted that Tris-borate buffer has a relatively large temperature-dependence of pH, whereas the other buffers do not exhibit major temperature-dependent pH changes. The imino proton regions of the ^1^H NMR spectra of the 89-nt RNA sample dissolved in each buffer are shown in Supplementary Figure S1. As expected, the RNA that was dissolved in the lower pH buffer exhibited more imino proton signals. The region derived from non-canonical base pairs, from 9.0 to 11.5 ppm, showed marked changes. Use of Tris-borate resulted in no signals in the non-canonical base-pairing region, whereas the use of MES or sodium phosphate gave at least four signals. Interestingly, the chemical shifts of the observed signals were almost identical in the range of pH 6–8 for the different buffers. These findings suggested that the structured region retained a common structure within the pH range, but that a lower pH induced more stable hydrogen bondings. As MES buffer was better suited for observing non-canonical signals, we decided to use MES-NaOH (pH 6.0) as a standard condition in this work. The 89-nt RNA sample dissolved into MES-NaOH (pH 5.5) yielded almost identical relative intensities of non-canonical and canonical signals compared with those from the sample dissolved in MES-NaOH (pH 6.0) (data not shown).

### icSHAPE library preparation

The *in vivo* click selective 2′-hydroxyl acylation and profiling experiment (icSHAPE) standard protocol was followed for library preparation and RNA secondary structure analysis (39). Briefly, HEK293T/17 cells were co-transfected with pEGFP-C2 and SINEUP-GFP. Twenty-four hours after transfection, cells were treated for in vivo SHAPE modification with NAI-N_3_ reagent (modified) or with dimethyl sulfoxide (DMSO) (mock control). Two biological replicates for each of the NAI-N_3_ and DMSO libraries were made. RNA was extracted by using an RNeasy Mini Kit (Qiagen, Hilden, Germany, Cat. no. 74106) and digested with DNase I to remove DNA contamination by using a TURBO DNA-free kit (Thermo Fisher Scientific, Cat. no. AM1907). Purified RNA was ribo-depleted by using a magnetic RiboZero kit (Illumina Inc., Hayward, CA, USA, Cat. no. MRZH11124), and RNA quality was checked with an Agilent RNA 6000 Nano kit (Agilent Technologies, Cat. no. 5067-1511). Next, 500 ng of ribo-depleted RNA was used for biotin-click reaction to label probed bases, and RNA was fragmented for 40 s by using RNA fragmentation reagents (Thermo Fisher Scientific, Cat. no. AM8740). Fragmented RNA was end-repaired, and the icSHAPE adapter ligation standard protocol was then followed. Adapter-ligated RNA was size-selected and reverse transcribed by using icSHAPE-specific barcoded RT-primer. (RT enzyme cannot pass through modified bases, and each RT stop signifies the position of a modified base.) In the next step, truncated cDNA products were selected by using a streptavidin-based system and further size-selected (39). Purified cDNA was circularized by using CircLigase II enzyme (Lucigen, Epicentre, Cat. no. CL9025K), and the library was amplified and quantified by using the standard protocol (34). Purified libraries were sequenced on a HiSeq2500 platform (first by using a 150-base single-end rapid mode, and then with a 100-base single-end high-output mode). The sequence details of all of the oligos used in the library preparation are described in the original protocol (39).

### icSHAPE sequence data analysis and secondary structure modeling

To ensure sufficient sequencing depth, the sequence data from rapid mode and high-output mode were merged together and a standard icSHAPE bioinformatics pipeline was used (39). In brief, after PCR duplicate removal and adapter trimming, raw sequencing data were mapped to human transcriptome (hg38) and a custom EGFP and SINEUP transcript index by using Bowtie2 (http://bowtie-bio.sourceforge.net/bowtie2/index.shtml). In the case of SINEUP, mapping was focused on the region starting from 1 bp upstream of SINE B2 and extending to 88 bp downstream of it to avoid normalization bias from flanking sequences and to capture reads uniquely mapping to SINE B2 only. Next, transcript abundance and RT stop numbers were calculated, and this was followed by calculation of the correlation between replicates and then replicate merging. The mean number of RT stops of the 90–95% most reactive bases was normalized to 1, and all other RT stops were scaled proportionally (at this step, for SINEUP, the tailing 88 nt were excluded from the normalization calculation). Enrichment reactivity scores were then calculated by subtracting the background signal (DMSO data) and outliers were removed by 90% Winsorization. Valid enrichment reactivity scores were filtered and visualized in IGV (Integrated Genome Viewer, http://software.broadinstitute.org/software/igv/home). The icSHAPE enrichment score data were used as a soft constraint in the RNAfold program of ViennaRNA (33). A linear mapping method was used to derive pairing probabilities, and the method described in reference (40) was selected to incorporate guiding pseudo-energies into the folding algorithm. The forna (force-directed RNA) server was used to draw the secondary structure (41).

## RESULTS

### Mutation and NMR analysis suggests that the structure of inverted SINE B2 is associated with its activity

**Figure 1.**
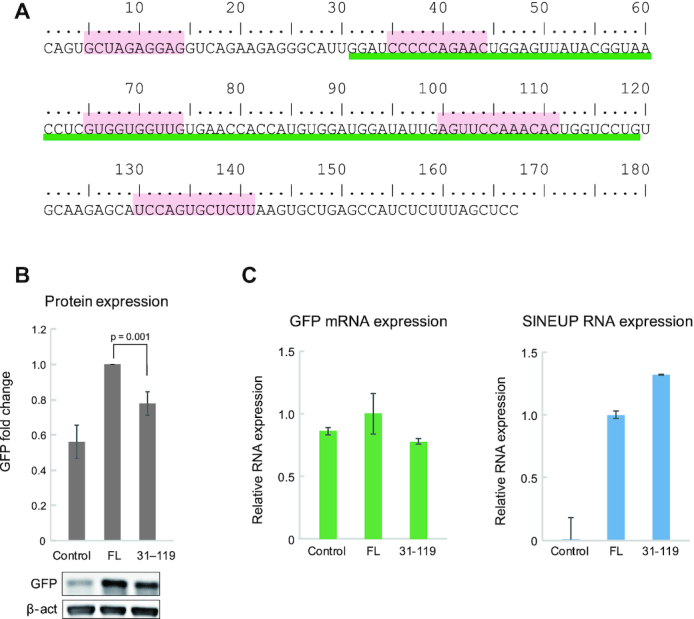
SINEUP activity analysis of fragmented inverted SINE B2. (**A**) The sequence of inverted SINE B2 embedded in AS-Uchl1 lncRNAs. Nucleotides 5–14, 35–44, 65–74, B-box (100–111) and A-box (130–141) are highlighted. Nucleotides 31–119 are underlined. (**B**) Western blot analysis of control, full-length (FL) and region 31–119 fragment. (**C**) qRT-PCR analysis of GFP mRNA and SINEUP-GFP RNA. Control: without SINEUP-GFP. *n* = 5. *P-*values were calculated by using a two-tailed Student's *t*-test; error bars indicate standard deviation.

First, we briefly outline the previously reported structure–function relationships of the inverted SINE B2 element. Figure 1A shows the RNA sequence of the 167-nt inverted SINE B2 element embedded in AS-Uchl1 lncRNAs that we examined here in our NMR study. In our previous report (19), three Gs were added at the 5′ end of the inverted SINE B2 for *in vitro* RNA transcription by T7 RNA polymerase and these artificial Gs were included in numbering; here only two Gs were added at the 5′ end, however for simplicity we eliminated them from the numbering and the nucleotide size (see Figure 1A). Therefore, it should be noted that nucleotide *n* in this work corresponds to the inverted SINE B2 embedded in AS-Uchl1 lncRNAs and to the nucleotide (*n* + 3) in ref. (19). Our previous NMR study of a fragment corresponding to nucleotide positions 56–93 of the inverted SINE B2 element suggested the formation of a unique stem-loop at positions 61–89; this was consistent with the secondary structure prediction using data from a chemical footprinting analysis (19). (See Figure 5G for the secondary structure prediction from ref. (19) using our numbering.) Deletion mutation analysis of the inverted SINE B2 indicates that the stem-loop in region 61–89 is essential for translation upregulation activity (19). However, as discussed later, the structure of a short RNA fragment is not generally consistent with that of the corresponding region in the case of lncRNAs, so that the accuracy of secondary structure prediction remains limited. Further deletion mutation analysis (nucleotides 5–14, 35–44, 65–74, 100–111 and 130–141, highlighted in pink in Figure 1A) showed that deletion of region 35–44 also caused SINEUP activity loss (Supplementary Figure S2, see ‘Materials and Methods’ section), and this was consistent with previous reported results (11). Chemical footprinting analysis suggests that region 35–40 is single stranded (19), but the structure of region 35–44 was not clarified in our previous NMR analysis.

Here, by modeling the SINEUP-GFP system, we further investigated whether the middle domain of the inverted SINE B2 structure without the 5′ and 3′ ends would retain SINEUP activity. Interestingly, a Δ1–30 Δ120–167 deletion mutant (containing only the 31–119 fragment) of the inverted SINE B2 (underlined in Figure 1A) retained about 80% of its SINEUP activity compared with the 167-nt full-length inverted SINE B2 (Figure 1B). Note that here we monitored whether GFP expression was altered by GFP mRNA expression and SINEUP RNA expression. RNA expression analysis demonstrated that GFP expression was not associated with expression of these RNAs (Figure 1C); this was consistent with previous reports (10–17). The result for GFP expression on nucleotides 31–119 suggests that neither the 5′ nor the 3′ end is crucial for SINEUP activity.

Next, we measured the NMR spectra of these mutants to identify the structured regions that were responsible for SINEUP activity. ^1^H NMR of imino protons acts as an excellent site-specific probe for base-pairing of RNAs (42,43). When ribonucleic acid makes a canonical base pair, two (UA pair) or three (GC pair) hydrogen bonds are formed. Each canonical base pair includes one hydrogen bonding between the imino group and acceptor (N3 of C and N1 of A). If hydrogen bonding occurs, the exchange of imino protons with solvent protons is suppressed; thus, imino protons can be detected as relatively sharp ^1^H NMR signals, indicating base-pairing of RNAs at specific locations.

**Figure 2.**
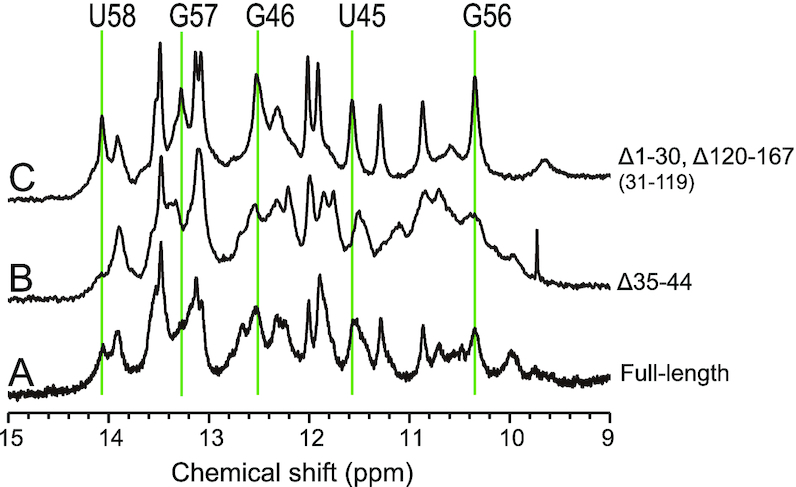
Imino regions of the 1D ^1^H NMR spectra of (**A**) FL-inverted SINE B2 (167 nt and GG), (**B**) the Δ35–44 mutant (157 nt and GG) and (**C**) the Δ1–30 Δ120–167 mutant (89 nt) in 20 mM MES-Na (pH 6.0) buffer with 50 mM NaCl at 298K. In the case of FLΔ35–44, U58, G57, G46, U45 and G56 signals were not observed.

Here, we compared the NMR spectra of the full-length inverted SINE B2 and its deletion mutants having different SINEUP activities. As outlined above, the Δ1–30 Δ120–167 deletion mutant (containing only the 31–119 fragment) of inverted SINE B2 showed SINEUP activity similar to that of full-length inverted SINE B2, whereas the Δ35–44 deletion mutant loses activity (11). To elucidate the structural differences among the two mutants and the full-length inverted SINE B2, we compared their ^1^H NMR imino resonances. Figure 2 shows the imino proton region of the 1D ^1^H NMR spectra of (A) full-length inverted SINE B2 (167 nt and GG), (B) the Δ35–44 mutant (157 nt and GG) and (C) the Δ1–30 Δ120–167 mutant (89 nt). The spectra showed numerous imino proton signals, which we used as the ‘fingerprints’ of the RNA structure. In comparing the spectra of the two mutants and the native full-length inverted SINE B2, we noticed that most of the signals showed identical chemical shifts. However, it was clear that five imino proton signals were not observed, or were substantially broadened, in the Δ35–44 mutant (Figure 2, trace B, solid lines). This indicates a lack of base-pairing for the regions, which introduces fast exchange of the imino protons with water protons and resultant loss of the ^1^H signals due to exchange broadening. As discussed below, these five changed or missing signals were assigned to the identifiers U58, G57, G46, U45 and G56 (solid lines in Figure 2). The data suggested that deletion of the 35–44 nt region, which led to a loss of SINEUP activity, caused breaking of the region 43–58 stem of the inverted SINE B2. In contrast, the 31–119 fragment exhibited these five signals (Figure 2, trace C). The results indicate that a specific structural change (absence or presence of the region 43–58 stem) may modulate SINEUP activity, as will be discussed further below.

### Domain survey of AS-Uchl1 inverted SINE B2 by NMR spectroscopy

To further explore the structure of inverted SINE B2, we next attempted to identify the structured domains of AS-Uchl1 inverted SINE B2. Because a functional stem-loop structure (SL1 in Figure 5G) was expected in the middle of the inverted SINE B2 (19), we first systematically deleted both ends of the full-length RNA in steps of 10 nt. We then measured the 1D ^1^H NMR spectra of these fragments and the full-length RNA (Supplementary Figure S3A). Analysis of the 1D ^1^H NMR spectra of the imino proton region acts as a sensitive probe to distinguish the formation of base pairs (i.e. secondary structures). If a fragment shows different imino ^1^H chemical shifts from those of the full-length RNA, then the secondary structure of the fragment should differ from that of the full-length RNA. This step allows us to survey regions (or domains) that can be safely divided without altering their structures. The ^1^H NMR spectra of fragments 11–157 (5′Δ10nt/3′Δ10nt) and 21–147 (5′Δ20nt/3′Δ20nt) did not show any signals that were not observed from the full-length RNA (Supplementary Figure S3A (a–c)). In contrast, shorter fragments such as 31–137 (5′Δ30nt/3′Δ30nt) and 41–127 (5′Δ40nt/3′Δ40nt) exhibited imino ^1^H signals different from those of the full-length RNA (see asterisks in Supplementary Figure S3A (d, e)); these were attributed to the formation of several artificial base pairs as a result of the deletions. This suggests that deletion of region 21–41 or 127–147, or both, induced a fold involving base-pairs distinct from those in the full-length (native) structure. We also successfully identified fragment 21–147 (5′Δ20nt/3′Δ20nt) as a region that retained the native structure.

**Figure 3.**
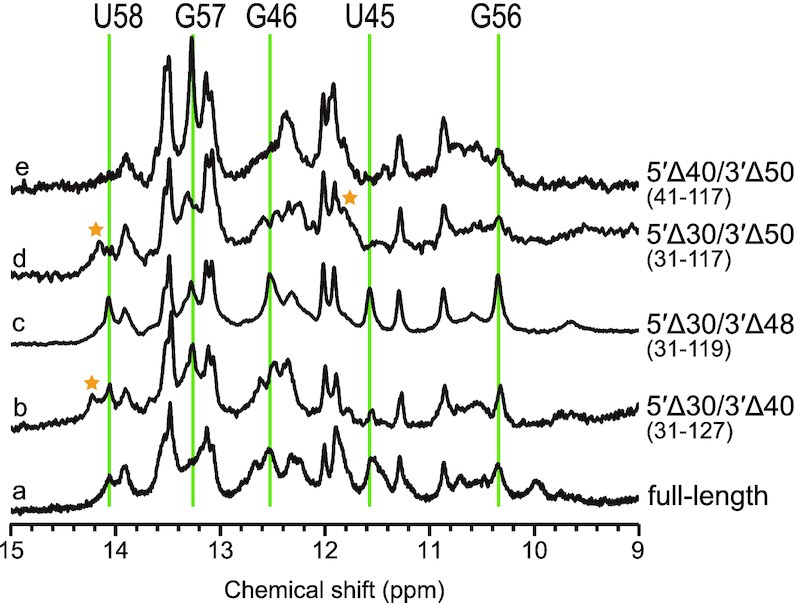
Imino regions of the 1D ^1^H NMR spectra of fragments and FL-inverted SINE B2 lncRNA. (a) FL, (b) 31–127 (5′ Δ30/3′ Δ40), (c) 31–119 (5′ Δ30/3′ Δ48), (d) 31–117 (5′ Δ30/3′ Δ50) and (e) 41–117 fragments (5′ Δ40/3′ Δ50). Signals not observed from FL are indicated by stars. Imino ^1^H signals that were not observed or broadened by deletion of the 5′- or 3′-end are indicated by solid lines. Spectra (a), and (c) had very similar features, suggesting the presence of a common structure.

To further identify the minimum region that reproduced the ^1^H NMR spectrum of the full-length RNA without inducing the formation of artificial base-pairs, we performed an NMR analysis of a series of asymmetric deletion fragments. Figure 3 shows the ^1^H 1D NMR spectra of (a) full-length inverted SINE B2 lncRNA, and fragments (b) 31–127 (5′Δ30nt/3′Δ40nt), (c) 31–119 (5′Δ30nt/3′Δ48nt), (d) 31–117 (5′Δ30nt/3′Δ50nt) and (e) 41–117 (5′Δ40nt/3′Δ50nt). Although the asymmetric deletion fragment 31–127 (5′Δ30nt/3′Δ40nt) in (b) still exhibited a signal that was not observed in the full-length RNA at 14.2 ppm in (a), to our surprise, it regained native signals (U58, G46) that were not observed in the symmetric deletion fragments 31–137 (5′Δ30nt/3′Δ30nt; Supplementary Figure S3d) and 41–127 (5′Δ40nt/3′Δ40nt; Supplementary Figure S3e). Interestingly, fragment 31–119 (5′Δ30nt/3′Δ48nt) in (c) showed all the signals of U45, G46, G56 and U58 without showing any artificial signals that were not observed in the full-length RNA (as suggested by Figure 2). This indicates that the 3′-end region may also influence the stability of the critical stem region at A43–U58, which is likely vital for the SINEUP activity of inverted SINE B2. This was further confirmed by a drastic change in the spectrum of fragment 31–117 (Figure 3 (d)), which nearly lost all the signals of U45, G46 and G56 (vertical lines). We checked several fragments that were shorter than fragment 31–119 but retained the 61–89 region; however, all such fragments gave signals that were not observed in the full-length RNA or lost the signals U45, G46, G56 or U58, or both (see Figure 3 (e) and Supplementary Figure S3B for examples). Thus, our 1D NMR-based survey allowed us to identify fragment 31–119 (5′Δ30nt/3′Δ48nt) as another region that retained the native structure of full-length inverted SINE B2. The drastic spectral changes caused by the deletion of a few residues suggest that this ‘divide-and-conquer’ approach would not be so straightforward for the determination of lncRNAs structures without the use of a careful systematic NMR-based survey, especially because their secondary and tertiary structures are largely unknown.

In summary, the full-length RNA could be shortened into at least two fragments (21–147 and 31–119) without substantially altering the core structural motif. The data suggested that full-length inverted SINE B2 is likely to have at least three structured regions: 21–31, 120–147 and 31–119. Fragment 21–147 did not give any artificial signals that were not observed in the full-length RNA, whereas fragment 31–137 had an artificial structure; this indicates that at least fragment 21–31 or fragment 137–147, or both, were required to maintain a native structure. Although the differences in the 1D spectra of fragments 21–147 and 31–119 in Supplementary Figure S3c and h) were not evident at this point, our NOE analysis of the fragment that included regions 21–31 and 120–147 (see below) clearly showed their stem structure. As indicated below, regions 1–21 and 141–167 were found to be dynamic (see Figure 5F for our NMR-based secondary structure); this is also evidenced by the minimal difference between the spectrum of the full-length RNA in (a) and that of fragment 21–147 in (c) in Supplementary Figure S3A.

### Computational prediction of the secondary structure of inverted SINE B2

**Figure 4.**
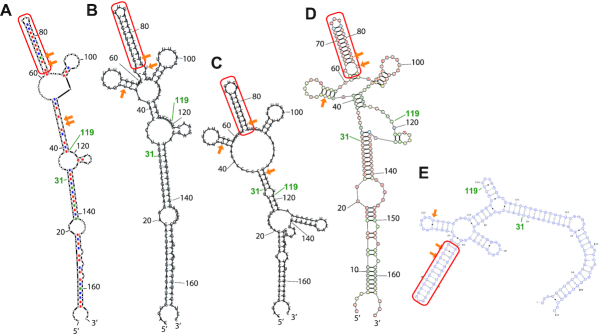
Schematic drawings of secondary structures of inverted SINE B2 predicted by computer programs. (**A**) Mfold, (**B**) RNAfold, (**C**) Vsfold5, (**D**) RNAstructure and (**E**) Vfold2D. The region G65 to U85 (circled by solid lines) was predicted as a stem-loop structure by all programs. Parameters for each program are described in the secondary structure prediction programs section of *‘*Materials and Methods’ section. Non-canonical base pairs (GU and UU pairs) formed between nucleotides 31 and 119 are indicated by arrows.

**Figure 5.**
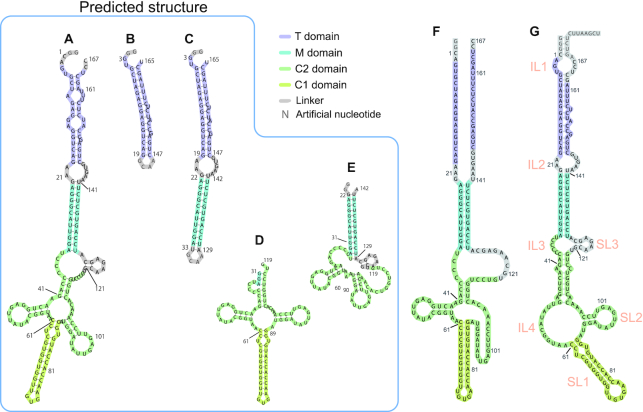
Schematic drawings of secondary structures of inverted SINE B2, based on secondary structure prediction by RNAfold. Inverted SINE B2 can be divided into four domains, namely T, M, C1 and C2. C1 is the same as SL1 (19). (**A**) FL, (**B**) T fragment, (**C**) TM fragment, (**D**) C fragment consisting of C1 and C2 domains and (**E**) MC2 fragment. (**F**) Schematic drawing of the secondary structure of FL-inverted SINE B2, as determined from our NMR data. (**G**) Schematic drawing of the secondary structure of FL inverted SINE B2, as determined from footprinting data (19). Nomenclature from reference (19) is shown in orange. Predicted structures (A) to (E) were calculated by using RNAfold. Parameters are described in the section on secondary structure prediction programs in ‘Materials and Methods’ section.

We next compared the domain structure derived from our NMR analysis with the computational predictions, which were used as reference structures. We checked five computational RNA secondary structure prediction programs, and in Figure 4 we list the resultant structures predicted by (A) Mfold, (B) RNAfold, (C) Vsfold5, (D) RNAstructure and (E) Vfold2D. All predictions were performed with the default (or recommended) parameters of each program, as described in the ‘Materials and Methods’ section. Despite the use of different free energy settings and different algorithms (such as pseudoknots in the Vsfold5 program), all algorithms predicted a stem-loop structure in the region around nucleotides 65–85 (Figure 4, circled by red line). The structure of a 38-nt fragment (56–93 region) including this region has been determined by NMR and shown to be essential for the regulatory function of SINEUPs (19). The other regions in fragment 31–119 showed dramatic variations among prediction programs. The numbers of non-canonical base pairs present in the 31–119 region also differed among programs (Table 1) and our NMR analysis. Examination of the ^1^H-^15^N HMQC (heteronuclear multiple quantum coherence) and ^1^H-^1^H NOESY NMR spectra revealed that region 31–119 included one UU pair and two GU pairs. The structures predicted by RNAfold and RNAstructure were consistent with our NMR experimental results (Table 1). We therefore used the RNAfold prediction as a primitive reference for the secondary structure (Figure 5A). In accordance with the results of the prediction, the region of each domain was rearranged. The presumably base-paired region involving nucleotides 3–19 and 147–165 was named the terminal domain (T), the region involving nucleotides 23–32 and 130–140 was named the middle domain (M), and region 34–119 was named the central domain (C). C consisted of two sub-domains: one, named C1, at nucleotides 61–89, was predicted to be a stable stem (SL1 in our previous report); the other, C2, was the remaining region (nucleotides 34–60 and 90–119) and included a GU pair. With secondary predictions by RNAfold in Figure 5 for the (B) T, (C) TM, (D) C and (E) MC2 domains used as references, we performed our NMR analysis (see Supplementary Figure S4 and Table 2). As discussed below, our NMR-based secondary structure (Figures 5F and 7A) demonstrated notable differences from those predicted for the full-length SINE B2 RNA or its domains in Figure 5A–E.

**Table 1.** Comparison of the number of non-canonical pairs in region 31–119, as observed in the NMR data, versus as predicted by various prediction programs

	# of GU	# of UU
NMR	2	1
Mfold	3	1
Vsfold5	3	0
RNAfold	2	1
Vfold2D	2	2
RNAStructure	2	1

The number of each non-canonical pair in RNAfold and RNAstructure were consistent with the NMR data.

**Table 2.** Assignment of imino proton signals of inverted SINE B2

		FL	MC2	TM	C
Residue	Domain	(ppm)	(ppm)	(ppm)	(ppm)
G24	M	12.52	12.50	12.54	
G25	M	10.68	10.65	10.71	
G26	M	13.15	13.12	13.18	
U29	M	11.51	11.47	11.53	
U30	M	13.43	13.36	13.41	
G31	M	11.82	11.73	11.80	
G32	M	12.25	12.20	12.29	
U34†				13.66	
G41	C2	12.32			
U45	C2	11.55	11.35		11.55
G46	C2	12.52			12.49
G56	C2	10.31	10.23		10.30
G57	C2	13.23	13.11		13.23
U58	C2	14.05			14.05
U63 or U87	C1	9.59			9.61
G65	C1	10.83			10.84
U66	C1	13.52			13.51
G67	C1	11.87			11.85
G68	C1	13.04			13.04
U69	C1	13.47			13.45
G70	C1	11.97			11.96
G71	C1	13.09			13.08
U72	C1	13.87			13.89
U85	C1	11.27			11.26
G86 or G88	C1	12.71			12.67
U87 or U63	C1	10.48			10.47
U112	C2	13.96			
G113	C2	13.19			
G114	C2	12.35			
U130	M	13.95	13.85	14.05	
G134	M	11.47	11.41	11.48	
U135	M	13.57	13.54	13.58	
G136	M	12.29	12.23	12.31	
U138	M	11.84	11.83	11.87	
U140	M	13.54	13.65	13.58	

U34† indicates artificial base pair with A in the linker at TM, showing as magenta cross at Supplementary Figure S4.

### Secondary structure determination of full-length inverted SINE B2

**Figure 6.**
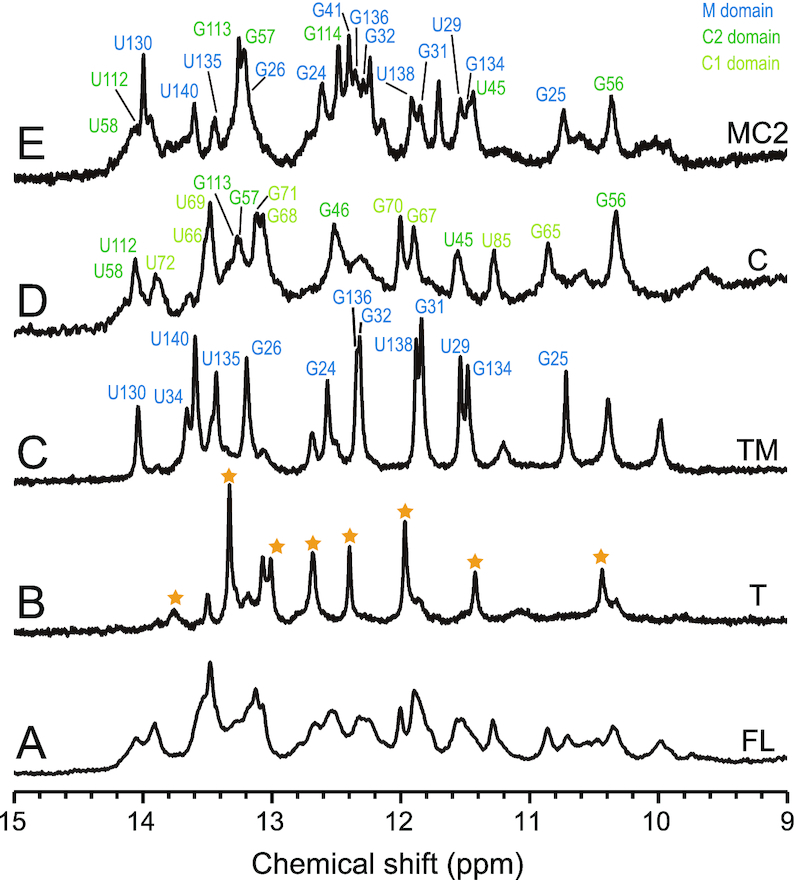
Imino proton regions of 1D ^1^H NMR spectra of full-length inverted SINE B2 (FL) and fragments. (**A**) FL, (**B**) T, (**C**) TM, (**D**) C (region 31–119) and (**E**) MC2 domains in 20 mM MES-Na (pH 6.0), 50 mM NaCl at 298K. Signal assignments are indicated by different colors (NOE assignments are given in Supplementary Figure S4 and Table 2) TM, MC2 and C exhibited signals that appeared at FL. The spectrum of the T domain showed signals that were not observed at FL (indicated by stars). These results suggested that the T domain did not have the stem structure predicted by the computational prediction programs.

For further NMR analysis of the structured domains, we prepared four inverted SINE B2 RNA fragments: domains T (nucleotides 1–19 and 147–167, connected by a GCAA linker), TM (nucleotides 1–34 and 129–167, connected by a GCAA linker), C (nucleotides 31–119) and MC2 (nucleotides 21–60 and 90–147). We then measured the 1D ^1^H NMR spectra of the imino protons of these four RNA fragments and compared them with the spectrum of the full-length RNA (Figure 6A).

Although the imino proton resonances of the TM, MC2 and C fragments (Figure 6C–E) generally agreed with those observed from the full-length RNA (Figure 6A), the spectrum for T (Figure 6B) differed greatly from that of the full-length RNA. In addition, TM did not exhibit artificial signals that were not observed from the full-length RNA, although none of the imino proton signals of the T fragment had the same chemical shifts as those of TM. As discussed later, all of the assigned imino signals for TM in Figure 6C are attributed to the M domain. These data suggest that the T domain of the full-length RNA or TM did not have the stem structure that was predicted by the computational programs.

**Figure 7.**
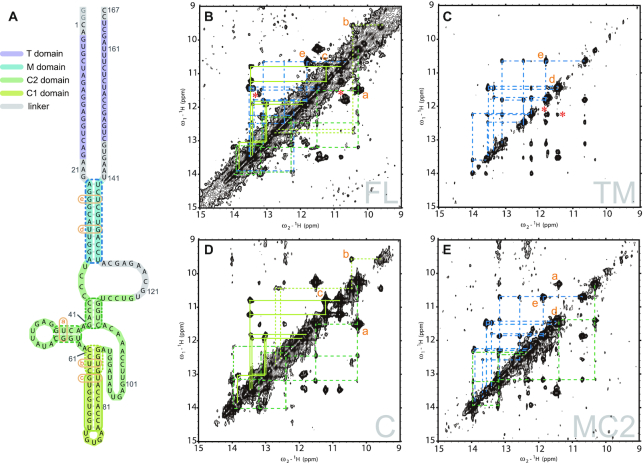
(**A**) Schematic drawing of the secondary structure of FL-inverted SINE B2, as determined from NMR data (identical to Figure 5F) for comparison with the 2D NMR data. Portions of the ^1^H-^1^H NOESY spectra of (**B**) FL, (**C**) TM fragment, (**D**) C fragment and (**E**) MC2 fragment. NOE connectivities are indicated by each corresponding color in A and B to E. Details of NOE assignments are given in Supplementary Figure S4. The chemical shift of each imino proton resonance is shown in Table 2. Intra-base-pair NOEs of non-canonical base pairs are indicated by orange letters. NOEs that have no corresponding peaks are indicated by red asterisks. The T region of FL (B) and the TM fragment (C) showed no NOE connectivities predicted by prediction programs, suggesting that the T domain did not form the predicted stem structure. The summation spectra of TM and C were almost consistent with that of FL with the exception of some NOEs (indicated by asterisks), as suggested by their 1D spectra.

We plotted the whole secondary structure of inverted SINE B2 (Figures 5F and 7A), as determined by the 2D NOESY analysis (see Supplementary Figure S4), which offers information on the pairing of specific bases through the nuclear Overhauser effect (NOE), as described below. We also show the imino region of the ^1^H-^1^H NOESY spectra of full-length inverted SINE B2 and the TM, MC2 and C fragments (Figure 7B–E; see also Supplementary Figure S4 for detailed assignments). Here, 2D NOESY connected the resonances of imino ^1^H for the base-paired nucleotides in spatial proximity. Thus, unlike in the case of chemical probing methods, these NOE contacts in the 2D NMR data allowed us to identify directly the canonical and non-canonical pairing of specific nucleotide bases for secondary structure determination. Most of the imino–imino NOEs of the M and C domains were assigned from the NMR data of TM, MC2 and C (Table 2). No NOE signals from the T domain, despite the prediction of its stem formation by the programs, were identified at TM and FL, suggesting that the region does not have a stable stem structure as predicted. Most of the signal positions of the TM, MC2 and C domains overlapped well with those of the full-length lncRNA (Figure 7B–E). These results suggested that we could successfully divide the full-length lncRNA into structured domains without altering the original structure. As shown in the examples in Figures 6 and 7, this finding is far from trivial without the guidance of NMR spectral data. In this way, we were able to assign the NOESY spectra of the full-length lncRNA (Figure 7B and Supplementary Figure S4A) by using the assignments of the optimally divided fragments as references. As indicated by the color-coded assignments, nearly all of the assigned NOE signals in the TM fragment were assigned to the M domain (cyan lines in Figure 7C; see assignments in Supplementary Figure S4C). All base pairs that were assigned to the M stem in the MC2 fragment (Figure 7E) also appeared in the TM fragment. Similarly, the signals of the base pairs assigned to the C2 domain (green dotted lines) in the C domain (Figure 7D) were consistent with those of the MC2 domain (Figure 7E). With the remaining signal assignments for the C1 domain (light green lines) in the C domain (Figure 7D), we were able to successfully complete assignments for nearly all the signals of the full-length inverted SINE B2 in Figure 7B.

Several weak, unassigned signals in TM may be attributable to the T domain. For example, two NOEs were unassigned in the TM fragment (red asterisks in Figure 7B, TM). The results above suggested that red-asterisked NOEs in the TM fragment were derived from the T domain, although these NOEs were not observed in the full-length lncRNA. Notably, all of the secondary structure prediction programs had strongly suggested that there was a stem structure in the T domain of the full-length lncRNA (see Figure 4). However, the NMR spectra of the TM fragment and the full-length lncRNA showed no NOE connectivity of the stem for the T domain that was predicted by those programs (See Supplementary Figure S4A and C). Likewise, although region 91–109 was strongly suggested by the secondary structure predictions to form a stem-loop structure (Figure 4), the imino–imino NOEs of these stems were observed only at temperatures below 288K. This result suggested that the region 91–109 stem was not stable, unlike the other stems in the full-length lncRNA.

In summary, our NMR-based study, for the first time, laid out a well-defined secondary structure and structural determinants for the intricate lncRNA element, 167-nt inverted SINE B2, for which the structural basis has until now been contradictory. In our previous result for the 38-nt fragment, a UU pair at the region 61–89 stem was not observed in the NMR spectra, although the structure calculated by simulated annealing showed the presence of a UU pair (19). In this report, a UU pair was clearly observed in the C domain and in the full-length lncRNA (Figure 7B, labeled b and Supplementary Figure S4A). Interestingly, four base pairs of the region 43–58 stem were not formed when the sequence ended before G118, even when A43 to G58 was included (Figure 3D and E; Supplementary Figure S3B). In the case of nucleotides 31–118, not only the region 43–56 stem (native structure) signals but also artificial signals were observed (Supplementary Figure S3g). In contrast, artificial signals were not observed in fragment 31–119 (Supplementary Figure S3h). These results indicated that residues U118 and G119 were likely required to form the region 43–58 stem-loop structure. Two NOESY cross peaks still remained unassigned in the full-length structure (red asterisks in Figure 7B and Supplementary Figure S4A), suggesting that these two NOEs may be due to inter-domain contacts. It may be therefore be possible to extract the tertiary structure of this system, as discussed below.

## DISCUSSION

Structural determination of functional RNA domains is essential to understanding their molecular architectures and ultimately their mechanisms of action, yet there have been technical challenges to defining the structures of long RNA molecules. Kaene *et al.* showed the tertiary structure of 155 nt RNA by introducing ^2^H-labeled segments effectively (25). Their structural determination of the target RNA core HIV-1 packaging signal is preceded by structural works on its smaller domains by several research groups in 2000s (44–47). Sashital *et al.* determined the structure of 111 nt U2/U6 snRNA (27). Barnwal *et al.* showed ‘divide-and-conquer’ method effectively in their structure determination of 68 nt CssA thermosensor RNA (29). Duszczyk *et al.* showed dimerization model of Xist A-repeat by using 26 nt A-repeat fragment (48). On the other hand, most of the NMR-based structural analyses of ncRNAs are limited to around 100 nt. Thus, no NMR-based tertiary structures of lncRNAs have been reported yet. There are only a few previous studies that defined lncRNA structures by other methods even at the secondary structure level with atomic resolution.

Here, we have successfully demonstrated that our NMR-based approach allows one to define a structure of the long ncRNA SINEUP by separating functional RNA sequences into workable smaller domains that largely retained the original structure and function. The whole secondary and higher order structures of inverted SINE B2 and other lncRNAs are largely unknown. Historically, RNA structures are predicted by physics-based free energy minimization, as exemplified by the prediction software that we used here. However, structure prediction algorithms for large RNA molecules with non-canonical interactions and pseudoknots are still limited in accuracy because of the vast conformational landscapes of RNA samples (49,50). Other secondary structure determination experiments, such as chemical probing, cannot provide information on the base-pairing of specific nucleotides or the stability of secondary structures, leaving the estimation of secondary structure formation—to a large extent—to the use of adopted prediction programs. Our findings here clearly demonstrate that our NMR-based approach is definitely amenable to the secondary structure determination of ∼200-nt structured RNAs that have little structural information. lncRNAs have a wide range of lengths, although functional regions (such as embedded SINE elements) often contain ∼200-nt structural domains consisting of several stem-loops. Secondary structure determination of such structured RNA domains is even more crucial than that of proteins, because RNA is capable not only of folding in multiple, different structures (which are often not functional), but also of folding by using artificial base-pairings that are not formed in native RNA. For instance, several functional RNAs can adopt two structures, such as riboswitches (51). The 1D ^1^H NMR spectra of the imino proton region were useful in our initial analysis of domain structure. Because chemical shifts of imino proton resonances are sensitively affected by secondary structure, we can eliminate fragments that would include artificial structures. At this point, it is unclear why the domain structures are so sensitive to the adjustment of the boundaries; our running hypothesis is that this may be related to the presence of multiple functional structures for the lncRNA. Our methods can be used to determine the secondary structures of target lncRNA elements directly, even when a region exhibits such conformational plasticity. However, in cases where the target lncRNA element has a single-domain structure, our strategy may have limitations. The structural details of many lncRNAs are still unknown, but structural similarity is expected among SINEUPs, a novel class of functional lncRNAs that upregulate protein expression. Because we showed here that inverted SINE B2 has a multi-domain structure, it is likely that NMR approach will be applicable to other important lncRNAs with multi-domain structures.

From a structure–function perspective on RNA domains, our approach based on NMR spectroscopy analysis offers advantages and the chance to understand the functional folding of ncRNAs. For example, our NMR experiments in Figure 2 clearly demonstrated that the region 35–44 deletion mutant that loses SINEUP activity also loses a stem structure at nucleotides 43–58, which was not predicted by previous chemical footprinting analyses. In contrast, fragment 31–119 retains the region 43–58 stem structure and 80% of SINEUP activity. These deletion NMR experiments indicated that preserving the region 43–58 stem-loop structure is likely to be vital for SINEUP function. Also, our NMR experiments suggested that fragment 31–119 retained a native fold, revealing that the region involving a majority of the C domain (nucleotides 33–121) is a primary structural determinant of the function of the lncRNA. Indeed, the NMR-based secondary structure shown in Figure 7 is tremendously different from any of the program-predicted structures in Figure 4, including that for the region 43–58 stem-loop structure.

Although ncRNAs are believed to function together with interacting molecules, such as RBPs (52), *in vitro* NMR measurements of ncRNAs without interacting molecules may yield RNA structures specific to the measurement conditions in a solution (similar to in cell condition), in the absence of other cellular components. For instance, to our surprise, our NMR experimental data indicated that the T domain and the region 91–109 stem-loop did not exhibit rigid stem structures, contrary to all of the secondary structure prediction results and the findings of previous studies (11,19). In fact, our analysis by icSHAPE showed high-enrichment-score protected regions in the T domain, further validating the presence of a single-strand region in this domain in living cells, whereas low-enrichment-score regions in the T domain were preferentially double-stranded as a result of the constraints of the RNAfold software (Supplementary Figure S5, see ‘Materials and Methods’ section) (38,53). Thus, our NMR results provide new insights into the RNA regions of inverted SINE B2, and the T domain dynamics may be applicable to binding proteins for SINEUP activity in living cells (54,55). They therefore emphasize the importance of experiment-based structural examination. It is notable that our NMR approach allowed us to assign the key NOE signals for an lncRNA element with a molar mass of 55 kDa and revealed previously unexpected structural features, including the indication of higher-order structure of the system. As a caveat, we note that future analysis and modeling of icSHAPE data may further shed light to possible differences between technologies and further refine our model. To this end, future analysis such as a comparison of *in vitro* and in cell structures will also be needed.

An understanding of the structures of lncRNAs is a crucial step toward understanding their cellular mechanisms. However, challenges with *in vitro* NMR structural analysis still remain, because RNAs adapt to different regulatory states by structural change across different cellular compartments (51). Because AS-Uchl1 RNAs and synthetic SINEUPs are intercellular mobile elements, nucleocytoplasmic shuttling happens after transcription (10,55). Therefore, different inverted SINE B2 structures may play central roles in the different regulatory states and in different cellular compartments. Whereas there are only minor differences in the C1 (SL1) domain (which is a stable structure) between our previous chemical footprinting and NMR study (19) and this NMR study, the C2 domains have quite different secondary structures (Figure 5F and G). We anticipate that the C2 structure regions may offer flexibility for differential regulation in various compartments, depending on whether the inverted SINE B2 is retained in the nucleus, transported by nucleocytoplasmic shuttling, or engaged in sense–antisense interaction and translation regulation on polysomes. Further studies are required to examine such possibilities.

Our NMR results further suggest that inverted SINE B2 forms a higher-order structure. There have been far fewer structural analyses of RNA by NMR and other methods compared with those of proteins, because the chemical and physical nature of RNAs makes them less suitable to the use of structural determination techniques, as described in the Introduction. Nevertheless, our NMR results (see Supplementary Figure S3F–H) demonstrate that the removal of U118 and G119 modulates the region 43–58 stem-loop structure, suggesting the possibility of long-range interactions between U118/G119 and the region 43–58 stem-loop. Imino proton signals of these residues are not currently assigned; therefore, the interaction between the region 43–58 stem-loop and U118/G119 is still unclear. The chemical footprinting results indicate that the regions of A43–G46 and U115–C117 are not likely single stranded (19) (Supplementary Figure S6A). Our current results suggested that the region 43–58 stem-loop may form a higher-order structure. In addition, our 2D NOESY data indicated that two unassigned NOEs were observable only in the full-length structure. These NOEs will be the target of our next structural analysis.

Determination of the tertiary structure of full-length inverted SINE B2 lncRNA will reveal more details of the molecular mechanism of SINEUP RNAs. Although fragment 31–119, which corresponds to the C domain of the full-length RNA, retained a native fold including the region 43–58 stem-loop structure, its activity was slightly weaker than that of the full-length RNA. As discussed above, our NMR data showed that the T domain in itself did not have rigid stem structure. A deletion experiment using a mutant of the full-length structure has shown that deletion of nucleotides 5–14, which correspond to the 5′ side of the T domain connected to the M domain, results in SINEUP activity similar to that of the full-length RNA, unlike in the region 35–44 deletion mutant (11). Considering that the M domain had a simple stem structure and that Δ1–30 Δ120–167 deletion mutant exhibited 80% of the SINEUP activity for the full-length (i.e. 20% loss), these results suggest that the 3′ side of T is more likely to interact with the C domain and to be involved in full activation of SINEUP function. As discussed above, the inverted SINE B2 lncRNA element is likely to have a stable tertiary structure at the C2 domain (long-range interaction between stem-loop of 43–58 and region 118–119) although region 90–110 of C2 domain did not form stable stem-loop; this was not predicted in previous studies. The next stage of our research will consist of determination of the full three-dimensional structure of SINE B2, including higher order structure, and an analysis of the relationship between structure and function. Such studies will not only offer valuable insights into the secondary structure of inverted SINE B2 but will also constitute an important stepping stone toward understanding its mechanisms of action. Finally, knowledge of the high-resolution structure of short structured domains, as determined by NMR, may allow us to identify key functional small structures, such as the k-mer recognition motifs of RBPs (56), which may be common folding components shared by different SINE sub-families, such as the mouse SINE B2 (10) and human Alu (14), that show SINEUP activity. SINEUPs have been used as RNA therapeutics to rescue disease caused by protein deficiency (15,57–58). The NMR approach presented here will increase our knowledge around the regulatory lncRNA structures of inverted SINE B2 and other SINEUPs. We are planning further structural analyses of other SINEs and SINEUPs in future. This knowledge is fundamental to understanding the functions of SINEUPs in specific cell types and cellular compartments, and further to optimizing the therapeutic applications of SINEUPs. The human transcriptome alone contains at least ∼28 000 lncRNAs (59); some 40% of tested lncRNAs seem to be functional in a single cell type and appear to have a variety of functions and likely mechanisms (60). Mapping their structural domains will play a fundamental role in our understanding of their regulatory roles and mechanisms.

## DATA AVAILABILITY

The icSHAPE data discussed in this publication have been deposited in NCBI’s Gene Expression Omnibus (61) and are accessible through GEO series accession number GSE146407 (https://www.ncbi.nlm.nih.gov/geo/query/acc.cgi?acc=GSE146407).

## Supplementary Material

gkaa598_Supplemental_File
