# Supplementary material for: An NMR-based approach reveals the core structure of the functional domain of SINEUP lncRNAs
Source: Nucleic Acids Res. 2020 Jul 22;48(16):9346–60. doi: 10.1093/nar/gkaa598 (PMC7498343; doi:10.1093/nar/gkaa598)
Supplement: gkaa598_Supplemental_File [file gkaa598_supplemental_file.pdf]

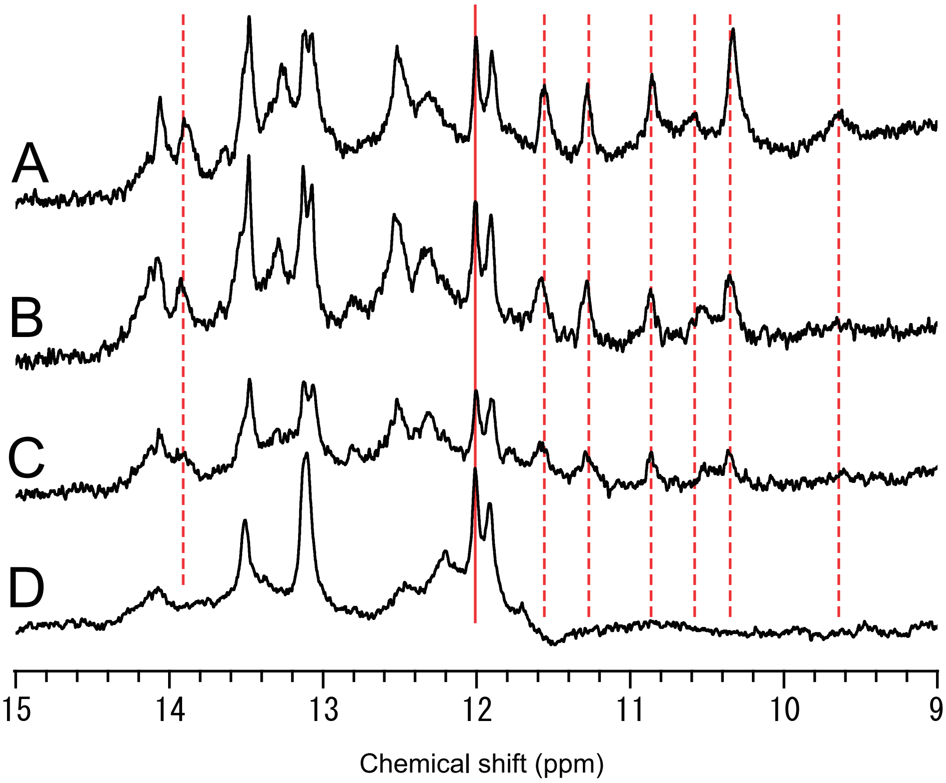

Supplementary Figure S1

^{1}H NMR spectra of nucleotides 31–119 in buffer with (A) 20 mM MES-Na(pH 6.0), (B) 20 mM sodium phosphate (pH 6.85), (C) 20 mM sodium phosphate (pH 7.2), and (D) 89 mM Tris-borate (pH 8.9). Buffers in (A) to (C) contained 50 mM NaCl and the samples were measured at 298K. Several signals changed in intensity with the changes in pH. At pH 6.0, the relative integration values of signals indicated by the red dashed lines were comparable (0.8 to 1.5) to that of the signal indicated by the red solid line, probably reflecting the changes in dynamics indicated by the red dashed lines. In contrast, few chemical shift changes were observed. This suggests that the structures were largely unchanged between conditions (A) and (D) for other regions. We selected condition (A) as the standard NMR condition used in this work to stabilize the dynamic region; such stabilization may be relevant to the activity of active site.

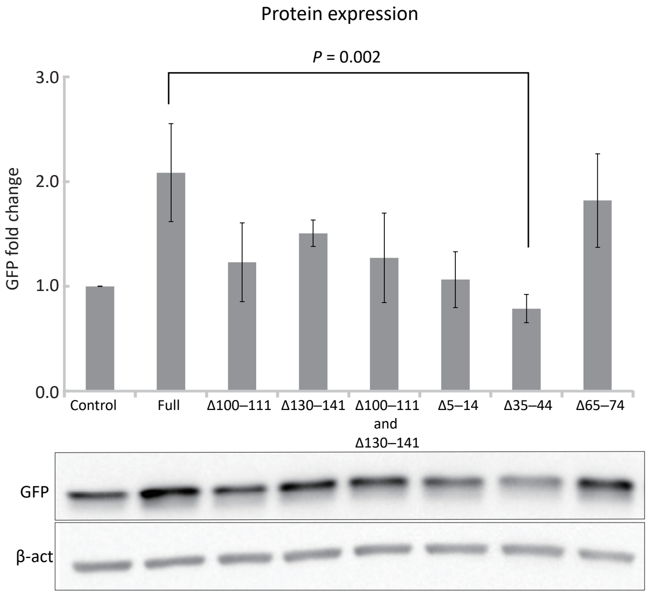

Supplementary Figure S2

SINEUP functional analysis of deletion mutations of inverted SINE B2. Western blot analysis of six deletion mutants. Control: without SINEUP-GFP. n = 3. *P* values were calculated by using a two-tailed Student's *t*-test; error bars indicate standard deviation.

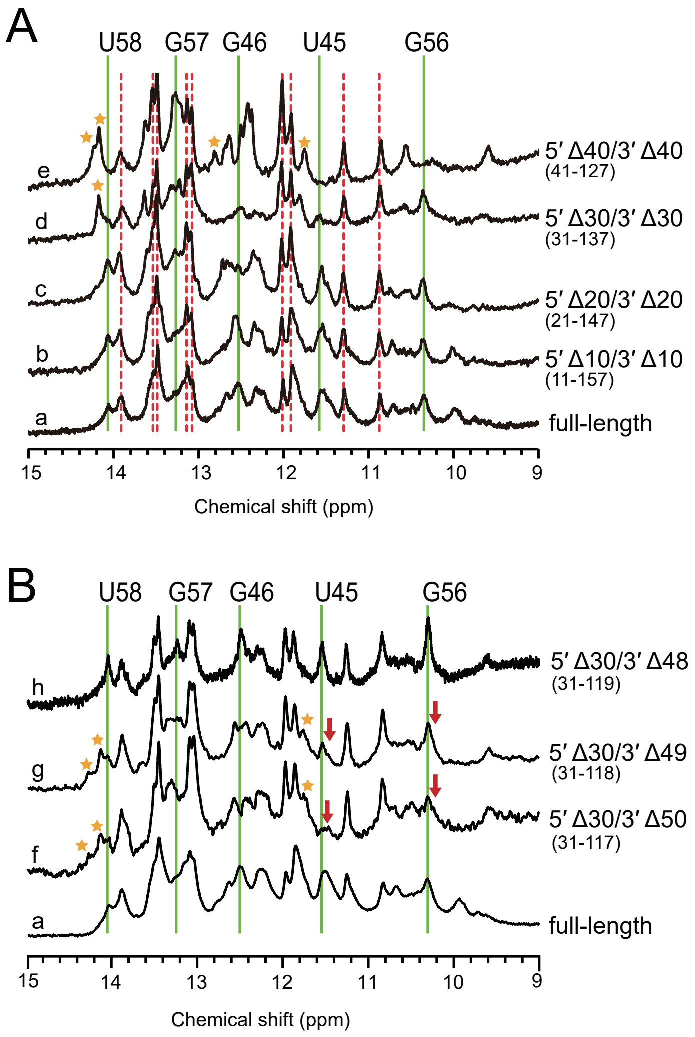

Supplementary Figure S3

Imino regions of the 1D ^{1}H NMR spectra of fragments and full-length (FL) inverted SINE B2. Panel A: (a) FL, (b) fragment 11–157, (c) 21–147, (d) 31–137, (e) 41–127. P anel B: (a) FL, (f) fragment 31–117, (g) fragment 31–118, (h) fragment 31–119. Signals that were not observed in the full-length SINE B2 are indicated by yellow asterisks. The signals that waken compared to FL and 31-119 are indicated by red arrow. Nine signals (indicated by the red dashed vertical lines) were exhibited by the full-length RNA and all fragments, suggesting that there was a stable stem in the G41 to A107 region, as based on the signal assignments shown in Supplementary Fig. S4. According to our previous work (19) identifying the formation of a stem-loop structure in the C61 to G89 region, including two non-canonical base pairs (SL1 region, Fig. 5G), we confirmed that the imino proton signals of this region showed almost identical chemical shifts, including a GU pair (Table 2). This suggests that the region forms a stable structure in full-length inverted SINE B2. Although fragments 31–137 and 41–127 in (d, e) exhibited several artificial signals (yellow asterisks) that were not observed in the full-length RNA, fragments 11–157 and 21–147 in (b, c) did not show any artificial signals that were not observed in the full-length RNA. This suggests that deletion of region 21–41 or 127–147, or both, induced a fold involving a partially artificial structure; this may be attributable to base-pairings distinct from those in the full-length (native) structure. Fragments 31–119 did not exhibit signals that were not observed in the full-length RNA. Eliminating nucleotides from the 3′-end of region 31–119 diminished signal intensities of the 43–58 stem (31–117 (f) and 31–118 (g), indicated by red arrow). This suggests that nucleotides G119 and U118 are involved in formation of the 43–58 stem structure.

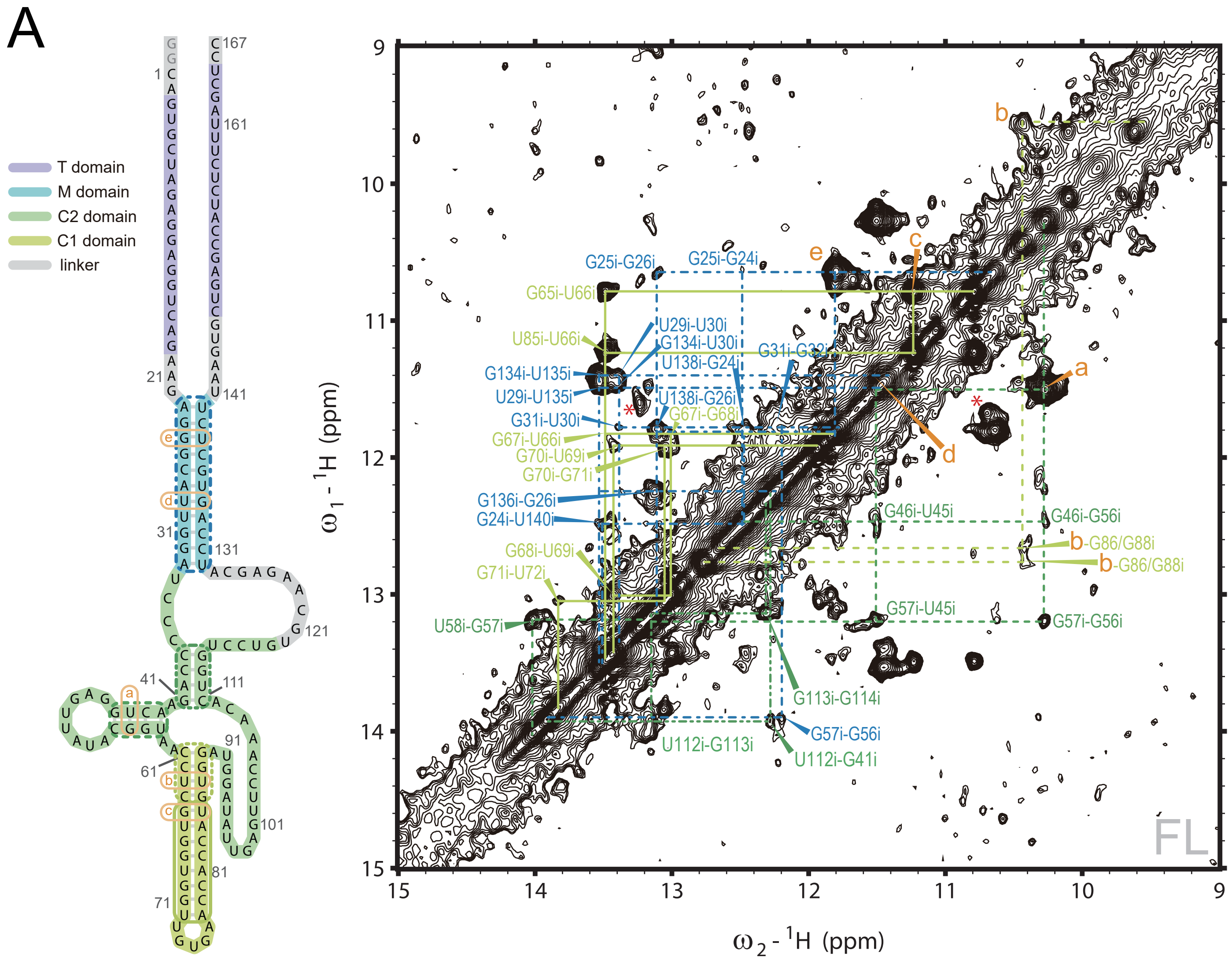

Supplementary Figure S4 A (continued)

B

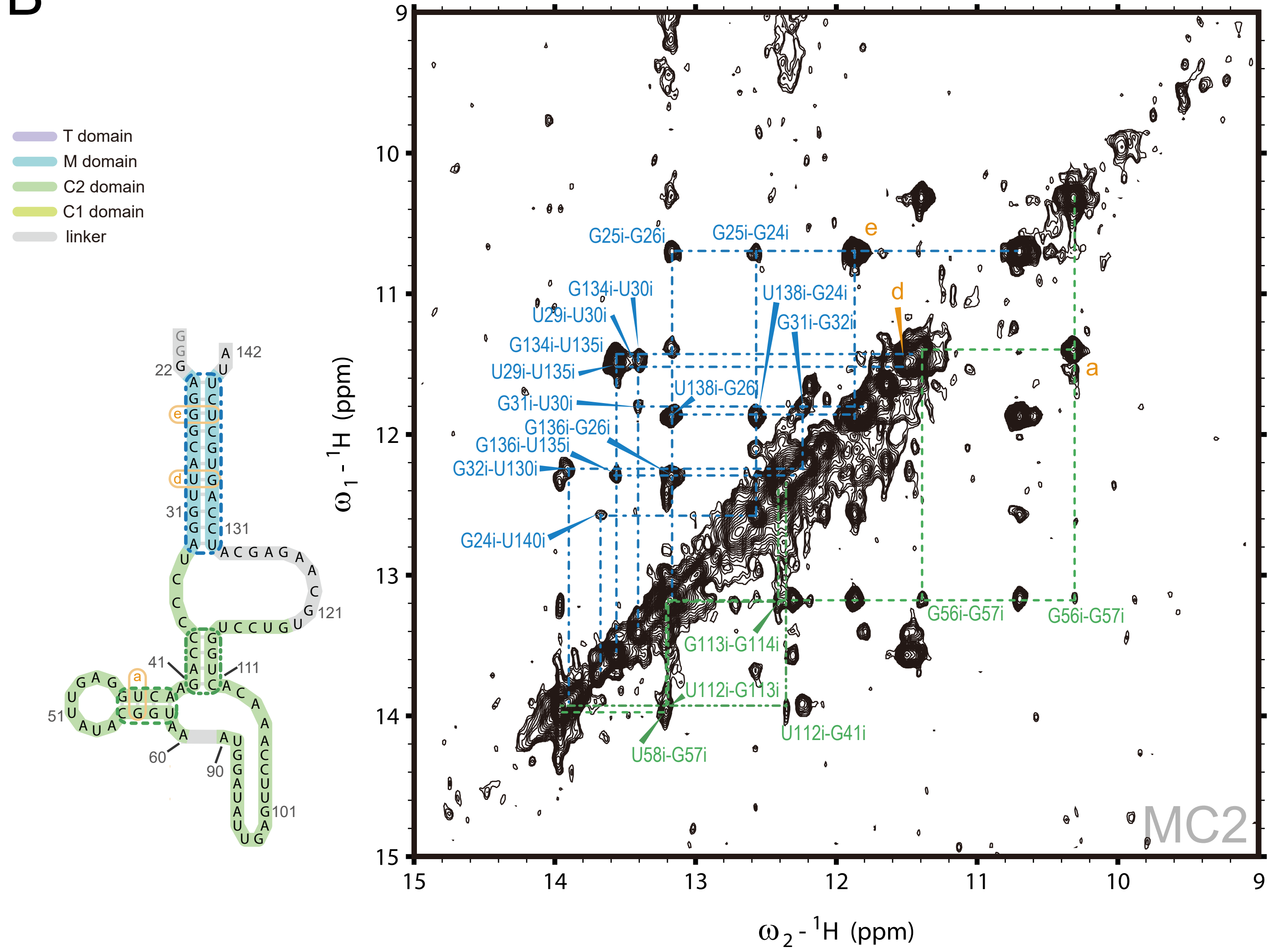

Supplementary Figure S4 B (continued)

C

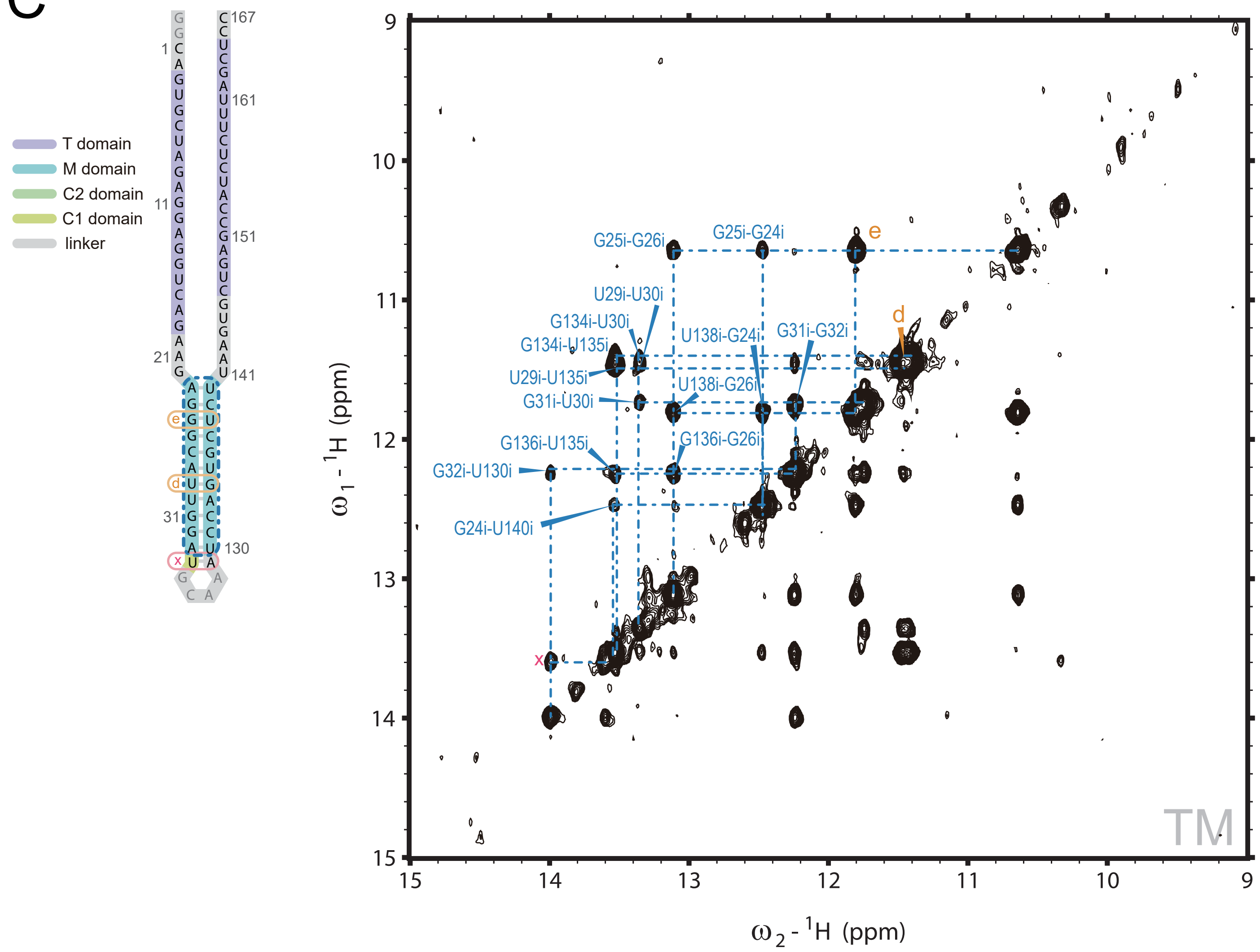

Supplementary Figure S4 C (continued)

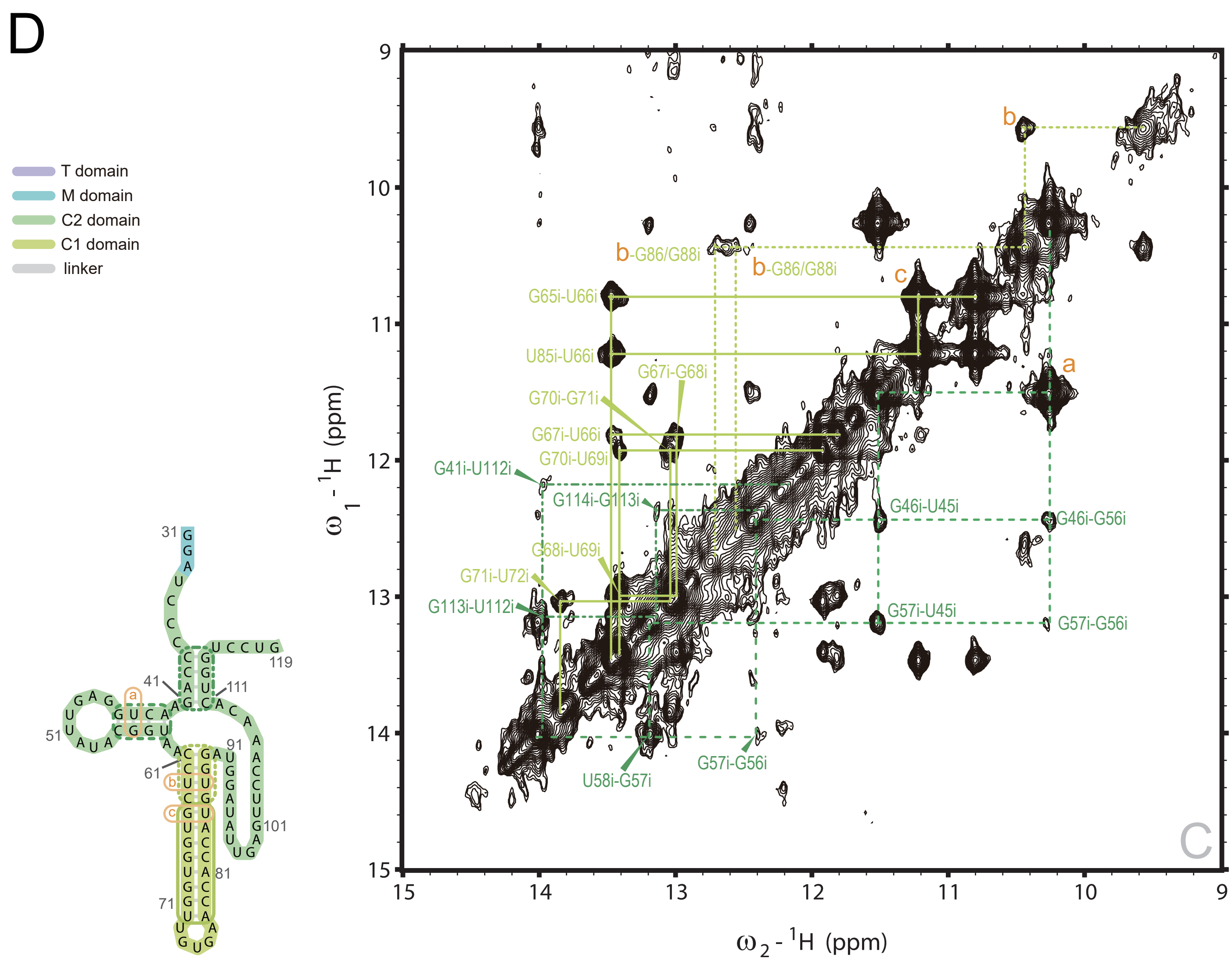

Supplementary Figure S4

Portions of 1H-1H NOESY spectra of (A) full-length inverted SINE B2, (B) MC2 fragment, (C) TM fragment, and (D) C fragment. Schematic drawings of each sequence are shown to the left of the spectra. NOE connectivities are indicated by each corresponding color in the schematic drawing of secondary structure. Intra-base pair NOEs of noncanonical base pairs are shown in orange. NOEs that were not assigned are indicated by red asterisks. An artificial base pair observed in the TM fragment is shown in magenta cross.

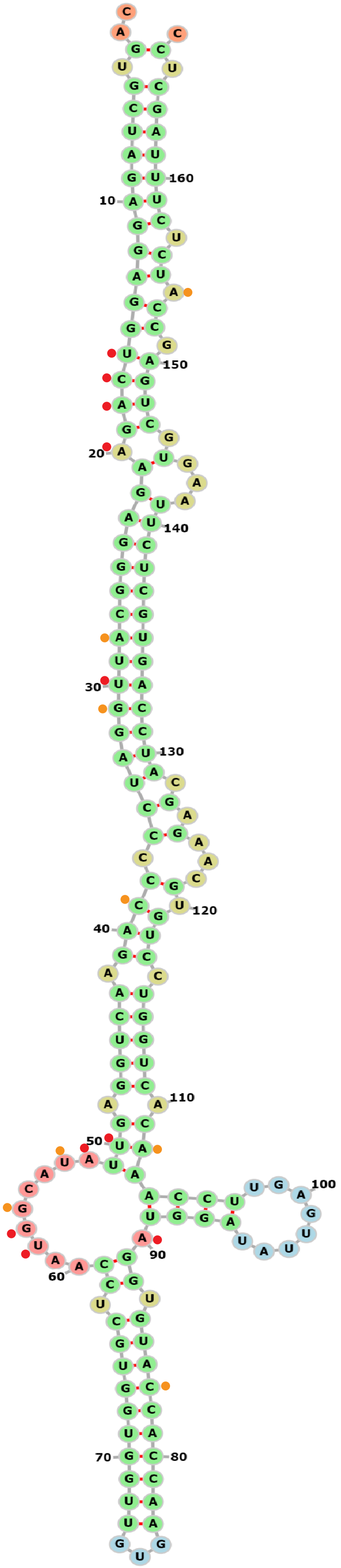

Supplementary Figure S5

icSHAPE data-driven in-cell secondary structure of the inverted SINE B2 region of SINEUP. The SINE B2 structure was probed by NAI-N3 reagent inside HEK293T/17 cells by using the icSHAPE protocol. icSHAPE enrichment score data were used as a soft constraint in the RNAfold program. Nucleotide color indicates the type of structure the nucleotide is in: green, stems (canonical helices); red, multiloops (junctions); yellow, interior loops; blue, hairpin loops; and orange, 5′ and 3′ unpaired regions. Red dots, icSHAPE enrichment score ≥ 0.85; orange dots, 0.85 > enrichment score ≥ 0.4; Enrichment scores are scaled on a 0 to 1 scale where 1 indicates a high probability of a nucleotide being in an unpaired state and 0 a tendency for it to be paired.

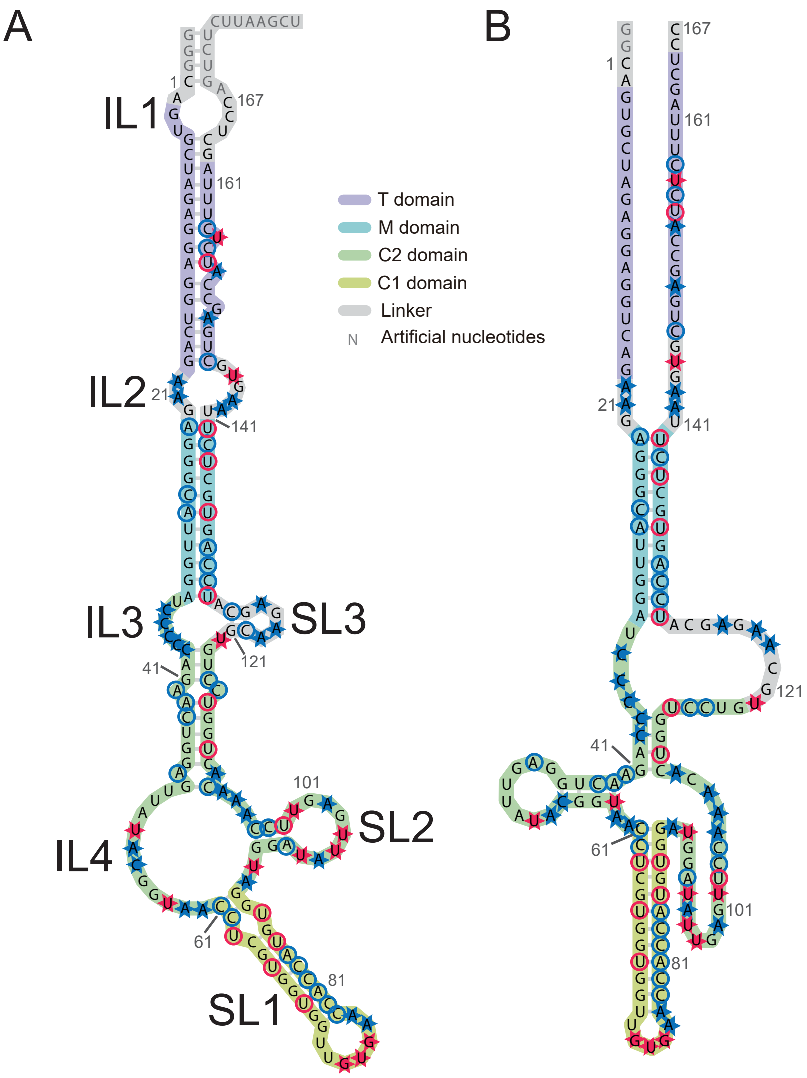

Supplementary Figure S6

(A) Schematic drawing of secondary structure determined by footprinting analysis (19). Dimethyl sulfate (DMS) and 1-cyclohexyl-(2-morpholinoethyl)carbodiimide metho-p-toluene sulfonate (CMCT) reactive nucleotides are indicated by blue and red stars, respectively, and non-reactive nucleotides are indicated by open circles. (B) Schematic drawing of secondary structure determined by NMR analysis. DMS and CMCT reactive and non-reactive nucleotides, as determined in reference (19), are indicated by the same symbols as in (A).
